# Genome-Based Analysis of Enterococcus faecium Bacteremia Associated with Recurrent and Mixed-Strain Infection

**DOI:** 10.1128/JCM.01520-17

**Published:** 2018-02-22

**Authors:** Kathy E. Raven, Theodore Gouliouris, Julian Parkhill, Sharon J. Peacock

**Affiliations:** a Department of Medicine, University of Cambridge, Cambridge, United Kingdom; b Public Health England, Clinical Microbiology and Public Health Laboratory, Addenbrooke's Hospital, Cambridge, United Kingdom; c Wellcome Trust Sanger Institute, Hinxton, Cambridge, United Kingdom; d London School of Hygiene and Tropical Medicine, London, United Kingdom; Johns Hopkins University School of Medicine

**Keywords:** VRE, mixed infection, recurrence

## Abstract

Vancomycin-resistant Enterococcus faecium (VREfm) bloodstream infections are associated with high recurrence rates. This study used genome sequencing to accurately distinguish the frequency of relapse and reinfection in patients with recurrent E. faecium bacteremia and to investigate strain relatedness in patients with apparent VREfm and vancomycin-susceptible E. faecium (VSEfm) mixed infection. A retrospective study was performed at the Cambridge University Hospitals NHS Foundation Trust (CUH) between November 2006 and December 2012. We analyzed the genomes of 44 E. faecium isolates from 21 patients (26 VREfm isolates from 12 patients with recurrent bacteremia and 18 isolates from 9 patients with putative VREfm/VSEfm mixed infection). Phenotypic antibiotic susceptibility was determined using a Vitek2 instrument. Genomes were compared with those of a further 263 E. faecium isolates associated with bacteremia in patients at CUH over the same time period. Pairwise comparison of core genomes indicated that 10 (71%) episodes of recurrent VREfm bacteremia were due to reinfection with a new strain, with reinfection being more likely with increasing time between the two positive cultures. The majority (78%) of patients with a mixed VREfm and VSEfm infection had unrelated strains. More than half (59%) of study isolates were closely related to another isolate associated with bacteremia from CUH. This included 60% of isolates associated with reinfection, indicating acquisition in the hospital. This study provides the first high-resolution insights into recurrence and mixed infection by E. faecium and demonstrates that reinfection with a new strain, often acquired from the hospital, is a driver of recurrence.

## INTRODUCTION

Enterococcus faecium is an important cause of bloodstream infections in critically ill and immunocompromised patients (1) and ranks among the 10 most common microorganisms associated with health care-associated infections in the United States (2). Bacteremia caused by vancomycin-resistant enterococci is associated with increased mortality, costs of care, and rates of recurrence compared to levels for vancomycin-susceptible strains (3–5).

Despite the establishment of linezolid and daptomycin as mainstay therapeutic agents for vancomycin-resistant E. faecium (VREfm) bacteremia since the early 2000s, recurrence remains a common clinical challenge, with rates ranging from 3% to 25% (6–8). Following apparent clearance of infection, recurrent bacteremia can be explained by either relapsing infection with the same strain due to a persistent focus of infection or reinfection with the same or a different strain. Differentiating between these scenarios is clinically relevant; relapsing infection requires investigation and interventions to deal with a persistent focus of infection, while reinfection is more likely to be associated with an underlying susceptibility that increases the risk of invasion due to breakdown of host immune defenses. Surprisingly little is known about the relative frequency of relapse versus reinfection in recurrent E. faecium bacteremia, with the literature limited to case reports (9–11) and case series predominantly limited to patients with Enterococcus faecalis infection (12, 13).

E. faecium bacteremia is polymicrobial in up to 35% of cases due to mixed infection with other bacterial genera or enterococcal species (14). Apparent mixed infection with VREfm and vancomycin-susceptible E. faecium (VSEfm) has been reported in the context of *in vivo* loss or gain of the *van* transposon by subpopulations of the same strain (15–17). However, while carriage of multiple strains of E. faecium is presumed to be common (18, 19), the frequency of mixed infection with different E. faecium strains is unknown.

Whole-genome sequencing has demonstrated discriminatory power superior to that of traditional bacterial typing techniques such as pulsed-field gel electrophoresis (PFGE) or multilocus sequence typing (MLST) in epidemiological investigations and in studies of the population structure of E. faecium causing bacteremia at local and national levels (20–23). Here, we use genome sequencing to gain a better understanding of E. faecium bacteremia. Specifically, we investigated strain relatedness in patients with recurrent VREfm bacteremia and in patients with apparent mixed bloodstream infection with VREfm and VSEfm. In addition, we compared the findings of genome sequencing with antibiotic resistance profiles.

## MATERIALS AND METHODS

A retrospective study was conducted at the Cambridge University Hospitals NHS Foundation Trust (CUH), a tertiary referral center in the United Kingdom with 1,170 beds and 350,000 occupied-bed-days per year. The rate of vancomycin resistance in E. faecium bacteremia isolates at CUH is high (>60%), approaching rates reported in the United States (2), and historical data from 2001 suggest that 32.6% of patients at CUH in high-risk wards carry VRE (either E. faecium or E. faecalis) (26).

All patients with VREfm bloodstream infection between November 2006 and December 2012 were identified using the diagnostic microbiology laboratory database. These cases were evaluated to identify all patients with (i) recurrence of VREfm bloodstream infection and/or (ii) putative mixed VREfm and VSEfm bloodstream infection. Recurrence was defined as a blood culture that was positive for VREfm taken >30 days after the index culture from a patient with intervening negative blood cultures and/or resolution of clinical signs of infection. Putative mixed VREfm and VSEfm infection was defined as the isolation of VREfm and VSEfm from the same blood culture or different cultures taken within 48 h of the index sample.

Fourteen patients fulfilled the criterion for recurrence, and 10 patients fulfilled the criterion for putative mixed VREfm and VSEfm infection, with no overlap of cases between the two groups. Seven of the patients had mixed infection with other bacterial species, as shown in Data Set S1 in the supplemental material. Cross-referencing these 24 patients with the bacterial freezer archive identified 44 isolates from 21 patients (12 patients with recurrence and 9 patients with mixed VREfm and VSEfm infection), who were the basis for this study. Clinical data for the 21 cases were collected from paper and computerized medical records using a standardized form, including the suspected focus of infection, underlying comorbidities, and dates of positive and negative blood cultures. Neutropenia was defined as a polymorphonuclear leukocyte count of less than 500 cells/μl within 24 h of the onset of bacteremia. The focus of infection was defined based on clinical, radiological, and microbiological features. Bacteremia was determined to be secondary to an intravascular device (i) if a positive intravascular catheter tip semiquantitative culture yielded more than 15 CFU of E. faecium with an antibiogram identical to that of the blood culture isolate (definite) or (ii) if no other focus of infection was identified in the presence of an intravascular catheter and/or if clinical signs of sepsis improved after line removal (probable). For neutropenic patients with no definite clinical focus, mucosal translocation was presumed to be the origin of the bacteremia, based on the definition from the Centers for Disease Control and Prevention (http://www.cdc.gov/nhsn/pdfs/pscmanual/4psc_clabscurrent.pdf). In cases of recurrent infection, a focus was considered persistent if there was an unresolved deep source of infection or if a potentially infected intravascular catheter was not removed between episodes of bacteremia. Ethical approval for the study was obtained from the local Research Ethics Committee (reference no. 13/EE/0044), and the need for informed consent was waived.

Twenty-one of the 44 isolates had been sequenced previously (27). For the 23 new E. faecium isolates sequenced here, bacteria were cultured on Columbia blood agar (CBA; Oxoid) for 48 h at 37**°**C in air. Phenotypic antimicrobial susceptibility testing for all 44 isolates was performed using a Vitek2 instrument (bioMérieux, Marcy l'Etoile, France) with an AST-P607 card. DNA was extracted using a QIAxtractor (Qiagen), and sequencing was performed on an Illumina HiSeq 2000 instrument. Sequence reads were assembled using Velvet and annotated using Prokka. The pangenome was estimated using Roary (28) with a 98% identification (ID) cutoff. The *van* gene in the two patients with genetically related VREfm and VSEfm isolates was extracted from the Roary pan genome and compared to the *vanA* gene extracted from a *vanA* transposon (NCBI accession number M97297) and the *vanB* gene extracted from Aus0004 (NCBI accession number CP003351) using BLAST. The presence of antibiotic resistance genes was determined using an in-house curated version of the ResFinder database (genes are listed in Data Set S3) (29) and ARIBA (https://github.com/sanger-pathogens/ariba/wiki).

Sequence data for an additional 263 E. faecium isolates associated with bloodstream infection in 263 patients at CUH between November 2006 and December 2012 and belonging to the hospital-adapted clone of clade A based on whole-genome sequence analysis were taken from Raven et al. (27). These 263 genomes together with the 44 study genomes were mapped to E. faecium Aus0004 (NCBI accession number CP003351) using SMALT (http://www.sanger.ac.uk/science/tools/smalt-0). Mobile genetic elements (identified based on annotation and PHAST [30]) and recombination events (identified using Gubbins [31]) were removed to identify the core genome. A maximum-likelihood tree was created using RAxML based on single nucleotide polymorphisms (SNPs) in the core genome. Pairwise SNP differences were calculated based on SNPs in the core genome.

### Accession number(s).

The sequences determined for this study were deposited in the European Nucleotide Archive under accession numbers ERR369962, ERR369975, ERR369979, ERR369980, ERR369983, ERR369992, ERR370001, ERR370025, ERR370028, ERR375298, ERR375306, ERR375310, ERR375319, ERR375322, ERR375348, ERR375405, ERR375413, ERR375440, ERR375455, ERR375476, ERR375478, ERR375492, ERR375503, ERR375511, ERR375534, ERR375548, ERR377424, ERR377426, ERR377429, ERR377430, ERR377432, ERR377434, ERR377441, ERR377443, ERR377453, ERR377456, ERR377495, ERR377498, ERR377503, ERR377512, ERR388704, ERR388709, ERR388720, and ERR388721 (see Data Set S1 in the supplemental material).

## RESULTS

**FIG 1:**
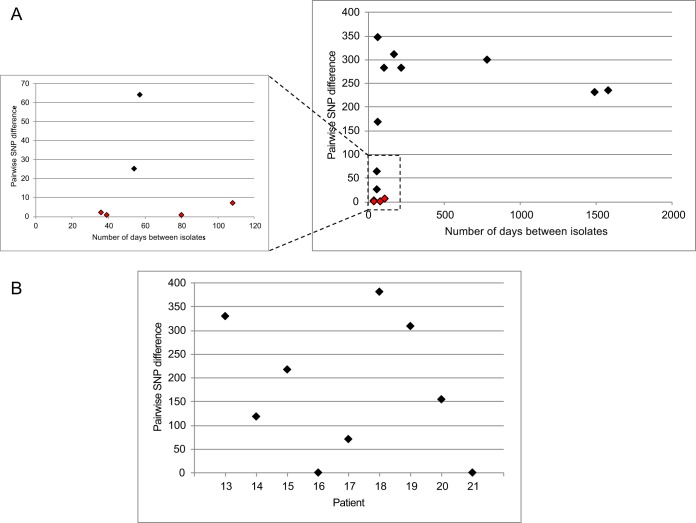
Genetic relatedness of isolates from the same patient with recurrent bacteremia or with mixed infection with VREfm and VSEfm. (A) The right-hand graph shows the pairwise core genome SNP difference between E. faecium isolates cultured from the same patient with recurrent bacteremia more than 30 days apart and the timing of the episodes. Red indicates isolate pairs that were closely related (1 to 7 SNPs) based on genome sequence data. The left-hand graph represents an expansion of the boxed area (under 100 SNPs) of the graph on the right. (B) Graph showing the pairwise core genome SNP difference between VREfm and VSEfm isolates cultured from the same patient with mixed infection within 48 h.

A retrospective review of patients at Cambridge University Hospitals NHS Foundation Trust (CUH) between November 2006 and December 2012 identified 231 patients with at least one episode of VREfm bacteremia. Of these, 14 patients had at least one episode of recurrence, giving an estimated recurrence rate of 6.1%. We identified 12 patients that had isolates from at least two episodes of VREfm bacteremia available for whole-genome sequencing (Table 1 summarizes patient data; see also Data Set S1 in the supplemental material for information on individual isolates). Ten patients had one recurrence, and two patients had two recurrences of bacteremia. To determine the genetic relatedness of isolates causing recurrence, we identified single nucleotide polymorphisms (SNPs) in the core genome based on mapping to a reference genome. Of the 14 isolate pairs associated with a recurrent bacteremia, four (from four patients) were closely related (1 to 7 SNPs; median, 1.5 SNPs) to the isolate from the previous episode (Fig. 1A and Table 1). This finding is highly indicative of relapse (or reinfection) with the same strain, based on a study that reported a genetic distance between E. faecium carried by the same person (within-host diversity) of six core SNPs (24). In contrast, 10 isolates (from 10 patients) were more genetically distant from the isolate from the previous episode (25 to 368 SNPs; median, 258 SNPs), which is consistent with reinfection by a different strain (Fig. 1A and Table 1). The two patients with two recurrences of bacteremia had both an episode of relapse (pairwise SNP difference of 2 and 7 SNPs, respectively) and an episode of reinfection with a new strain (25 and 309 SNPs, respectively) (Table 1). The SNPs acquired between the first and second isolate for the four genetically related isolate pairs were located in different genes in different patients (Table S1). The median time to first recurrence across the study population was 80 days (range, 39 to 1,578 days), and the second episodes of recurrence occurred 36 and 168 days after the preceding bacteremia. Comparison of the timing of recurrence with the genomic analyses indicated that all isolates from cases of relapse/reinfection with the same strain were isolated within 108 days of each other, while recurrences due to reinfection with a different strain were equally likely to occur within 108 days (5/10 episodes) and after 108 days (5/10).

**TABLE 1:** Clinical and isolate details for the patient cohort

Infection type and patient no.	Age (yr)^*a*^	Gender	Comorbidity(ies)^*b*^	No. of isolates	Presumptive source of infection by episode^*c*^			Year of first isolate	No. of days between episodes		No. of SNPs between episodes^*d*^			Interpretation
					1st	2nd	3rd		1st to 2nd	2nd to 3rd	1st to 2nd	2nd to 3rd	1st to 3rd	
Recurrence														
1	67	Male	SOM	3	Urinary	Urinary	Urinary	2010	54	36	25	2	25	Reinfection/relapse
2	50	Male	HM, SCT	2	MT/IV	MT/IV		2007	1,578		234			Reinfection
3^*e*^	56	Male	HM	2	MT/IV	MT/IV		2009	61		268			Reinfection
4^*e*^	24	Female	Congenital neutropenia	3	IV	MT/IV	MT/IV	2012	108	168	7	311	309	Relapse/reinfection
5	44	Female	SOT, ESRD, DM	2	IV	Unknown		2009	777		299			Reinfection
6	42	Female	Alcoholic liver disease	2	IV	IV		2009	215		282			Reinfection
7	13	Male	HM, SCT, ESRD	2	MT/IV	IV		2007	1,484		232			Reinfection
8	41	Female	HM, SCT, ESRD	2	MT/IV	MT/IV		2011	57		64			Reinfection
9	10	Female	HM	2	IV	MT/IV		2010	80		1			Relapse
10	0	Male	HM	2	IV	MT/IV		2010	59		348			Reinfection
11	15	Female	HM	2	MT/IV	MT/IV		2010	39		1			Relapse
12	39	Male	HM	2	MT/IV	MT/IV		2012	104		282			Reinfection
Mixed VREfm-VSEfm														
13	59	Female	SOM, HM	2	IV			2012	2		329			Genetically distinct
14	72	Male	SOM, HM	2	MT			2012	0		119			Genetically distinct
15	62	Male	HM, SCT	2	IV			2011	0		217			Genetically distinct
16	50	Male	HM, SCT	2	MT/IV			2009	0		0			Genetically related
17	56	Male	HM	2	IV			2008	0		18			Genetically distinct
18	59	Male	SOM	2	IA (biliary)			2008	0		381			Genetically distinct
19	63	Male	HM, SOT	2	IV			2009	1		203			Genetically distinct
20	19	Male	HM, SCT	2	MT/IV			2010	0		154			Genetically distinct
21	48	Female	ESRD, LC	2	Lung, IA			2010	0		0			Genetically related

a Age at the time of first bacteremia.

b Comorbidities identified across all episodes of bacteremia in the study (see Data set S1 in the supplemental material for a breakdown by bacteremic episode). SOM, solid organ malignancy; HM, hematological malignancy; SCT, stem cell transplant; SOT, solid organ transplant; ESRD, end stage renal disease; DM, diabetes mellitus; LC, liver cirrhosis.

c MT, mucosal translocation; IV, intravascular; IA, intra-abdominal; MT/IV, mucosal translocation with possible concurrent intravascular catheter infection.

d Number of SNPs based on mapping to a reference genome (E. faecium Aus0004).

e Patients with a recurrence with a different strain for whom a central venous catheter was retained between episodes of bacteremia.

All 12 study patients with recurrent bacteremia had multiple comorbidities that predisposed to VREfm bacteremia (Table 1 and Data Set S1). The most probable source for the bacteremia was defined for each case (Table 1). Mucosal translocation (*n* = 7, with possible concurrent intravascular catheter infection) and intravascular catheters (*n* = 5) were the most common sources of bacteremia for the study patients. There was no clear difference identified between the sources of infection in patients with recurrence due to the same or different strains (Tables 1 and S2). The four episodes of recurrence due to the same strain were associated with presumed persistent intravenous catheter colonization and/or gut carriage (*n* = 3) or with failure to eradicate a persistent focus of infection (*n* = 1, chronic pyelonephritis associated with kidney stones) (Tables 1 and S2). A central venous catheter was known to be retained between episodes of bacteremia in 2/10 cases with reinfection with a different strain, meaning that whole-genome sequencing was able to refute these as being a persistent focus.

**FIG 2:**
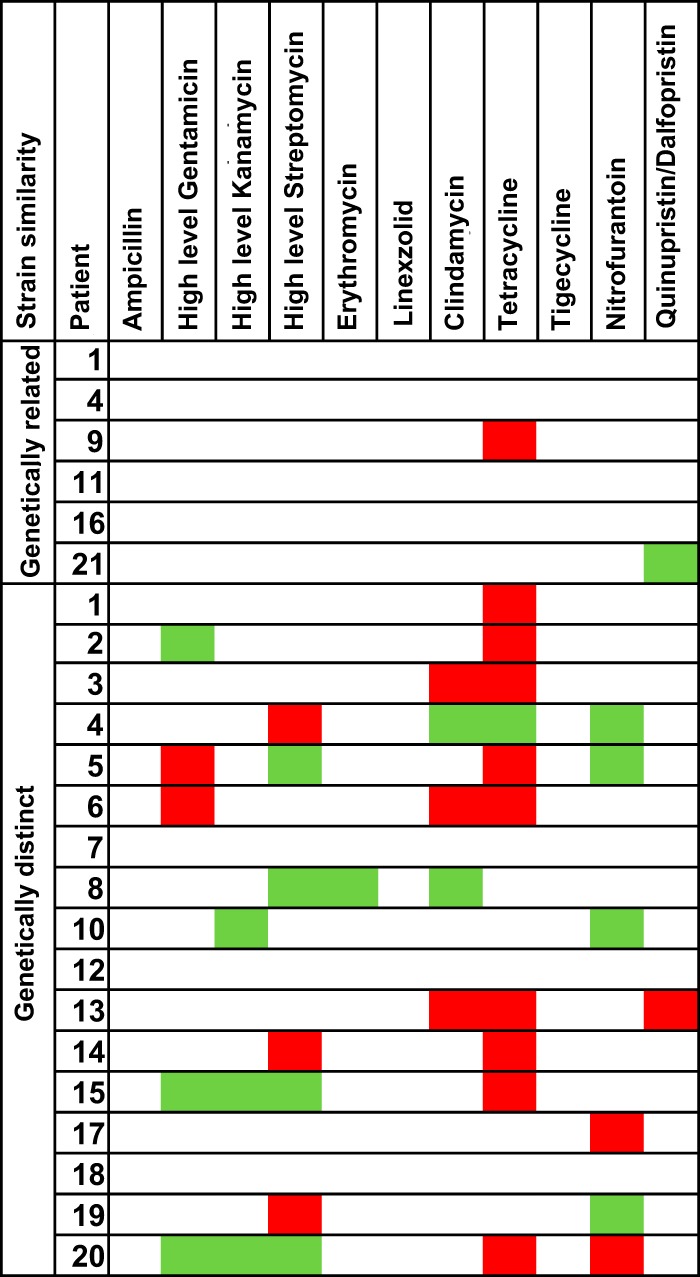
Comparison of strain similarities based on genome sequence data and antibiograms. Two patients with both relapse and reinfection occur twice in the list. Changes in antibiotic resistance between isolates from the same patient are shown (red, change from susceptible to resistant; green, change from resistant to susceptible; white, no change).

The retrospective review of CUH patients also identified nine patients with putative mixed bloodstream infection with VREfm and VSEfm for whom both isolates were available for whole-genome sequencing (Table 1). A pairwise core genome comparison of the 9 VREfm/VSEfm pairs revealed that 7/9 (78%) patients had isolate pairs that were genetically distinct (median, 217 SNPs; range, 70 to 381 SNPs) (Fig. 1B), which is consistent with true mixed-strain infection. The most common source of infection for patients with true mixed infections was an intravascular catheter (4/7, or 57%). The remaining two patients had isolates that were identical at the core genome level. Further analysis of the genetic content between these two pairs through comparison to the ResFinder database confirmed the variable presence of the *vanRSHAXYZ* genes, which encode vancomycin resistance. There was insufficient sequence adjacent to the *vanA* transposon in the genome assemblies to identify the genetic location of these genes, so differences in gene content between the VREfm and VSEfm isolates in each pair were assessed. In one isolate pair (from patient 16) the *van* genes had been lost together with 21 genes, including 7 genes best matched to a plasmid (based on a BLAST search), suggesting that they may have been lost/gained together with part of a plasmid (Data Set S2). Two genes labeled as *tetM* and *ermB* were lost alongside the *van* genes in this patient, but both isolates retained a copy of *tetM* and *ermB*, and so this may not have affected the wider antibiotic resistance phenotype (Fig. 2). In the second isolate pair (from patient 21) an additional 14 genes had been lost with the *van* genes, including five genes located adjacent in the genome (Data Set S2), suggesting that *vanA* was not gained/lost as part of a plasmid but may have moved as part of a smaller transposable element.

We evaluated whether the phenotypic antibiotic resistance profile (antibiogram) to 11 drugs (excluding glycopeptides) could be used to distinguish between genetically related and distinct strains from the same patient (Fig. 2). Of the six isolate pairs that were closely related in the two study collections, four had identical antibiograms, one varied by tetracycline resistance associated with gain/loss of the *tetM* gene, and one varied between susceptible and intermediate resistance to quinupristin-dalfopristin (Fig. 2). Of the 17 strain pairs that were genetically different, three had identical antibiograms, and the remainder had between 1 and 5 (median, 2) differences (Fig. 2). The most variable antibiotic was tetracycline (10/17 pairs), followed by high-level resistance to streptomycin (*n* = 7) and nitrofurantoin (*n* = 6). Since 3/7 identical antibiograms (43%) belonged to genetically distinct strains, this suggests that an antibiogram does not reliably distinguish between genetically related and distinct strains from the same patient. However, more than one change in the antibiogram was identified only in genetically distinct strains (12/17 genetically distinct pairs).

The high rate of true mixed VREfm and VSEfm infection and of recurrence with a new strain indicates carriage of multiple lineages or the acquisition of new strains over time. Health care settings are associated with the acquisition of E. faecium, and so we investigated CUH as a putative source by combining the 44 study E. faecium genomes with an additional 263 E. faecium genomes associated with bloodstream infection in 263 patients at the same hospital over the same time period (2006 to 2012) (Fig. S1). Based on analysis of the combined genomes, 26 of the 44 study isolates were closely related to at least one CUH isolate (0 to 8 SNPs; median, 3), including 3 isolates that were closely related to an isolate from another study patient. This included isolates from 6/7 patients infected with different VREfm and VSEfm strains and 6/10 patients with recurrence caused by different strains. The remaining isolates were between 12 and 86 SNPs (median, 33 SNPs) from the closest genetic match.

## DISCUSSION

This study represents the first use of whole-genome sequencing in the context of E. faecium bacteremia to investigate the relative rates of relapse and reinfection in recurrent infections and to study mixed infection with VREfm and VSEfm. Although rates of recurrence vary in the literature, the estimated rate of 6.1% identified at CUH is within the range of rates reported previously (6–8).

We found that the majority of patients in our study had a recurrent VREfm bacteremia caused by reinfection with a new strain. This finding supports that of Cheng et al. who found reinfection to be responsible for ∼70% of recurrence based on PFGE although that study focused primarily on E. faecalis (12). These reinfections could be due either to persistent carriage of a genetically distinct strain or to reinfection with a newly acquired strain. We found that at least 60% of reinfections were caused by isolates that were genetically closely related to another bacteremia isolate from CUH, suggesting cross-transmission in the hospital. Additionally, the rates of cross-transmission found in this study are likely to be an underestimate since asymptomatic gut carriage and the environment represent large reservoirs of VREfm and were not sampled in this study. These findings suggest that the emphasis on preventing recurrent VREfm bacteremia should be on infection control and minimizing periods of susceptibility to infection. Further studies will be required to elucidate the role of the environment, staff, and patients as sources for these hospital acquisitions to improve infection control.

The results of this study suggest that recurrence with the same strain may be related to time. Episodes of recurrence with the same strain were identified only up to 108 days apart, which concurs with findings by Baran et al. based on E. faecalis and E. faecium isolates from a total of three patients (13). In contrast to our findings for same-strain recurrence, our study showed that bacteremic episodes due to reinfections with a distinct strain were roughly equally likely to occur within and after 108 days of each other and as early as 57 days apart. Further work will be required using larger sample sizes from multiple centers to determine whether there is a true relationship between the relatedness of E. faecium strains causing recurrence and the time between episodes.

We also determined that the majority of patients with mixed VREfm and VSEfm bacteremia were infected with two genetically distinct strains. This result differs from the finding by Cardenas et al. that four patients had closely related VREfm and VSEfm strains associated with bacteremia based on MLST (16). The true mixed infections in our study frequently varied in antibiotic resistance profiles. While most cases of mixed VREfm and VSEfm infection would be detected during routine disc susceptibility testing, this variation in antibiotic resistance profiles could complicate treatment in cases that go undetected. Although the numbers are low in our study, it was interesting that true mixed VREfm and VSEfm infections were commonly suspected to originate from an intravascular source, suggesting that central venous catheters may become colonized with multiple strains of E. faecium.

The results of our study suggest that antibiograms lack accuracy in predicting the genetic relatedness of strains. The utility of antibiograms for determining the relatedness of E. faecium strains has not previously been evaluated, but our finding that a pair of identical strains could vary in their resistance to antibiotics is consistent with the fact that E. faecium has a highly mobile genome, with many resistance genes carried on mobile genetic elements.

Our study may have implications for future evaluation of VREfm treatment efficacy. There are currently no randomized controlled trials to define the optimal antibiotic for the treatment of VREfm bacteremia. Current knowledge is based on retrospective observational studies comparing linezolid to daptomycin, where recurrent infection is often defined as one of the outcome measures in the absence of bacterial typing results (6, 8, 25). These studies imply that early recurrence (often assessed at 30 or 60 days after treatment completion) is caused by true relapse (6, 8) or rely on phenotype, such as identical antibiograms, to infer relapse (25). Our results show that in the absence of prospective randomized studies or bacterial genotyping, one needs to question whether a recurrent infection is indeed due to ineffective therapy and not due to underlying, confounding patient-related factors conferring increased susceptibility to reinfection. Future studies should address this issue.

This study has several limitations. We did not sequence multiple colonies from the same sample to assess diversity, meaning that apparent reinfections could have been mixed infections at the outset. The study samples were retrieved from frozen stock, and it is not possible to know whether these were originally created from a single or multiple colonies. It is not possible to differentiate between relapse and reinfection by the same strain, introducing an element of uncertainty into our classification. The true level of mixed infection will be higher than we report here since we assessed only patients with a VREfm and VSEfm mixed infection and analyzed one colony of each from every bacteremic episode. Finally, the rate of recurrence identified here may be an underestimate since repeat cultures were not taken systematically.

In conclusion, the findings of this study show that the majority of VREfm recurrences and mixed VREfm and VSEfm infections are due to different strains and that antibiograms lack accuracy in determining genetic relatedness. This observation has important implications for infection control as it highlights the importance of reducing cross-transmission in vulnerable patient groups.

## Supplementary Material

Supplemental material
