# Supplementary material for: Genome-Based Analysis of Enterococcus faecium Bacteremia Associated with Recurrent and Mixed-Strain Infection
Source: J Clin Microbiol. 2018 Feb 22;56(3):e01520-17. doi: 10.1128/JCM.01520-17 (PMC5824064; doi:10.1128/JCM.01520-17)
Supplement: Supplemental material [file supp_56_3_e01520-17__index.html]

Supplemental material 

# Genome-Based Analysis of Enterococcus faecium Bacteremia Associated with Recurrent and Mixed-Strain Infection

## Supplemental material

- Supplemental file 1 -

  Fig. S1 (Relationship of study isolates to the CUH *E. faecium* bacteremia population) and Tables S1 (Details of SNP locations in the second isolate compared to those in the first isolate for genetically related isolate pairs) and S2 (Clinical details for the patient cohort and summary of results)

  PDF, 131K
- Supplemental file 2 -

  Data Sets S1 (Clinical details for each episode of bacteremia), S2 (Gene differences between VREfm and VSEfm isolates in patients with mixed VREfm and VSEfm bacteremias), and S3 (List of genes in curated ResFinder database)

  XLSX, 84K
