# Supplementary material for: Genome-Based Analysis of Enterococcus faecium Bacteremia Associated with Recurrent and Mixed-Strain Infection
Source: J Clin Microbiol. 2018 Feb 22;56(3):e01520-17. doi: 10.1128/JCM.01520-17 (PMC5824064; doi:10.1128/JCM.01520-17)
Supplement: Supplemental material [file JCM.01520-17_zjm999095848s1.pdf]

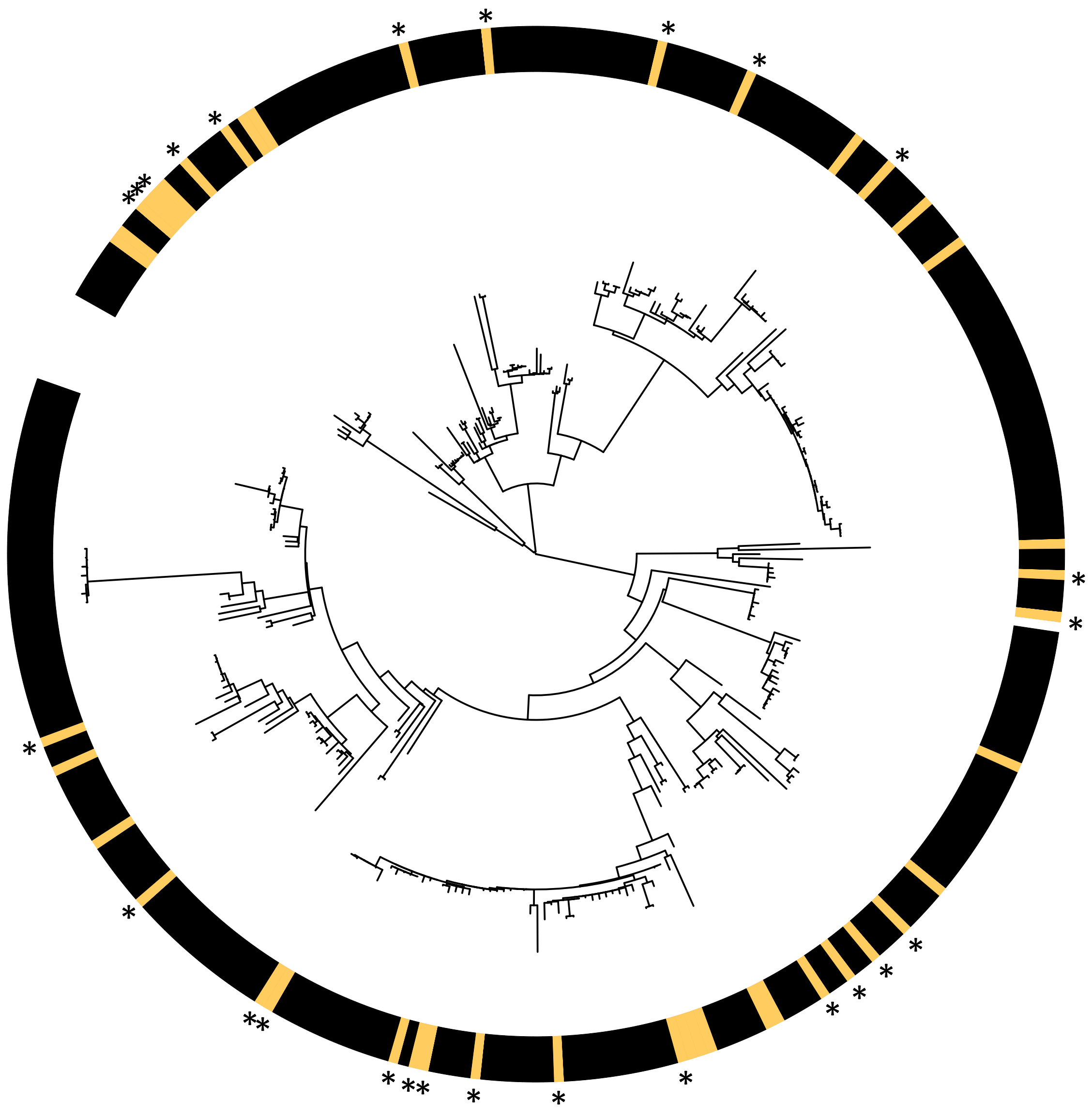

Figure S1. Relationship of study isolates to the CUH *E. faecium* bacteremia population. Maximum-likelihood tree based on SNPs in the core genome of the 44 study isolates and 263 additional CUH isolates associated with bacteremia. Colored ring shows the collection (orange = study collection, black = additional isolates from patients at CUH with bacteremia, white = reference strain (*E. faecium* Aus0004)), asterisks indicate a study isolate that was closely related to another CUH isolate.

Patient	SNP location in Aus0004 genome	Gene number in Aus0004 genome	Annotation in Aus0004 genome
1	1382437	prfC	peptide chain release factor 3
	541476	EFAU004_00525	sensor histidine kinase
4	526113	Not applicable	Intergenic
	805395	EFAU004_00773	hypothetical protein
	1127830	EFAU004_01101	sensor histidine kinase
	1999869	EFAU004_01976	sugar transferase
	2278684	EFAU004_02249	C4-dicarboxylate anaerobic carrier
	2505112	EFAU004_02487	Snf2 family protein
	2655579	Not applicable	Intergenic
9	2541224	EFAU004_02519	adenine deaminase
11	1270840	EFAU004_01239	acetyl-CoA acetyltransferase/hydroxymethylglutaryl-CoA reductase, degradative

Table S1. Details of SNP locations in the second isolate compared to the first isolate for genetically related isolate pairs (excluding VSEfm and VREfm pairs that are 0 SNPs different)

	Recurrence (12 patients, 14 recurrences)		Mixed infection (9 patients)	
**Age at index case, median (range)**	42 (0-67)		59 (19-72)	
**Gender, male**	6 (50%)		7 (78%)	
**Comorbidities**				
Malignancy	9 (75%)		8 (89%)	
Immunosuppression	10 (83%)		7 (78%)	
End-stage renal disease	1 (8%)▪		1 (11%)	
**Isolate details**	**Same strain (n=4)**	**Different strain (n=10)**	**Same strain (n=2)**	**Different strain (n=7)**
**Time interval in days, median (range)**	59.5 (36-108)	136 (54-1578)	0	0 (0-2)
**Same antibiogram**	3 (75%)	2 (20%)	2 (100%)	1 (17%)
**Clinical focus of first bacteremia**				
Intra-abdominal	0	0	1 (50%)	1 (17%)
Intravenous catheter	2 (50%)	3 (30%)*	0	4 (57%)
Mucosal translocation♦	1 (25%)	6 (60%)	1 (50%)	2 (33%)
Urinary	1 (25%)	1 (10%)	0	0
**Suspected persistent focus (possible, definite)**	2 (50%), 1 (25%)	2 (20%), 1 (10%)	N/A	N/A

▪ Two further patients developed end-stage renal disease at the time of recurrence
♦ Intravenous catheter infection was also possible but not definite in all but one case
* One patient had concomitant septic thrombophlebitis
All values expressed as number (%) unless otherwise specified.

Table S2. Clinical details for the patient cohort and summary of results. Top: Clinical demographics for 9 patients with mixed infection and 12 with recurrence. Bottom: Isolate details. For recurrence, the numbers shown for time and antibiogram are for each episode of bacteremia compared to the previous episode of bacteremia (therefore patients can appear in both the ‘same strain’ and ‘different strain’ columns in cases with two recurrences of bacteremia), and the focus of infection is given for the first episode of bacteremia.
